# How circulating tumor cluster biology contributes to the metastatic cascade: from invasion to dissemination and dormancy

**DOI:** 10.1007/s10555-023-10124-z

**Published:** 2023-07-13

**Authors:** Mostafa M Nasr, Conor C Lynch

**Affiliations:** 1 Tumor Biology Department, H. Lee Moffitt Cancer Center and Research Institute, Tampa, FL 33612 USA; 2 Cancer Biology Ph.D. Program, University of South Florida, Tampa, FL USA

**Keywords:** Metastasis, Circulating tumor cells, Circulating tumor clusters, Cancer dissemination, Cancer dormancy, Tumor microenvironment

## Abstract

Circulating tumor cells (CTCs) are known to be prognostic for metastatic relapse and are detected in patients as solitary cells or cell clusters. Circulating tumor cell clusters (CTC clusters) have been observed clinically for decades and are of significantly higher metastatic potential compared to solitary CTCs. Recent studies suggest distinct differences in CTC cluster biology regarding invasion and survival in circulation. However, differences regarding dissemination, dormancy, and reawakening require more investigations compared to solitary CTCs. Here, we review the current state of CTC cluster research and consider their clinical significance. In addition, we discuss the concept of collective invasion by CTC clusters and molecular evidence as to how cluster survival in circulation compares to that of solitary CTCs. Molecular differences between solitary and clustered CTCs during dormancy and reawakening programs will also be discussed. We also highlight future directions to advance our current understanding of CTC cluster biology.

## History and clinical significance of CTC clusters

The first detection of solitary circulating tumor cells (CTCs) in cancer patient’s peripheral blood was made by Thomas Ashworth in 1869 [1, 2]. However, clinical detection of CTC clusters has only been noted in the last two decades with the emergence of improved technologies [3, 4]. In both breast and prostate cancer, CTC clusters have been detected in patient peripheral blood at low frequency compared to solitary CTCs [5, 6]. Moreover, CTC cluster “emboli” could also be detected in the lung microvasculature of patients with metastatic breast cancer or metastatic cervical carcinoma [7]. With the development of improved isolation and detection methods of CTCs using highly sensitive microfluidic systems [5, 8, 9], the detection of CTC clusters has been noted in most cancer types including, but not limited to, lung cancer [10–14], prostate cancer [6, 15–18], breast cancer [13, 16, 19–22], colorectal cancer [23], liver cancer [24], pancreatic cancer [16, 25], melanoma [19, 26], and gastric cancer [27]. In pre-clinical models, the first evidence of CTC clusters was noted as early as 1954 [28]. Subsequently, many studies have shown the existence of these “aggregates” metastasizing to the lungs and livers of animal models [3, 29–31]. These observations led to the intriguing question as to whether CTC clusters would confer advantages in metastatic potential compared to solitary CTCs. To this end, studies have shown that CTC clusters not only possess higher metastatic potential than solitary CTCs but are also independent predictors of poor patient survival and prognosis [5, 18, 32–35]. However, our current molecular understanding of CTC clusters in terms of metastatic potential is far from complete.

## Distinctive characteristics of CTC clusters

**Fig. 1:**
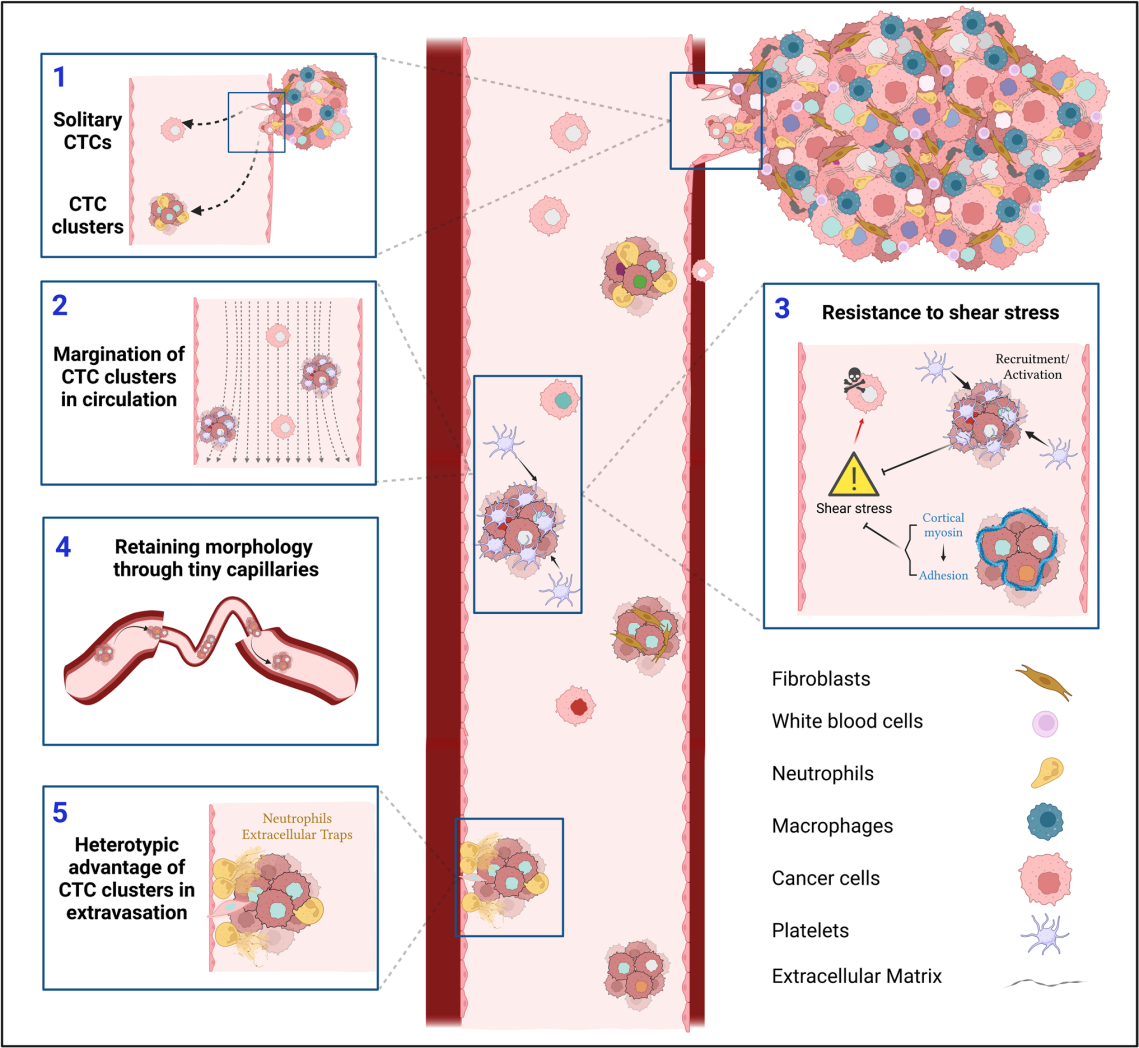
Distinct advantages of CTC clusters during the metastatic cascade. (1) Intravasation of cancer cells as solitary cells or clusters. (2) CTC clusters are bigger in size, so they travel slower and closer to the endothelium allowing for quicker extravasation. (3) CTC clusters are more resistant to shear stress compared to solitary CTCs through cellular (recruiting platelets) and molecular differences (reshaping cortical dynamics of myosin). (4) CTC clusters are structurally dynamic and can adapt their morphology in tiny capillaries. (5) CTC clusters can bind to other cells like neutrophils enabling extravasation through neutrophil extracellular traps

CTC cluster composition can range from approximately 2–45 cells [3]. Early studies showed that CTC clusters are less apoptotic than solitary CTCs suggesting a role for cell-cell adhesion in protecting the cancer cells from *anoikis* during the circulation [36, 37]. Different CTCs isolated from lung cancer patients (*n* = 2) show apoptotic cells (evident by fragmented and condensed DAPI stained nuclear morphology) in solitary CTCs, while little to no evidence of apoptosis was detected in CTC clusters [36]. Similar findings were reported in an *in vivo* model of head and neck squamous cell carcinoma (HNSCC) suggesting that CTC clusters are more resistant to *anoikis* [38]. Cluster formation may also provide protection against the shear stress and turbulent fluid dynamics encountered during arterial blood flow. For example, CTC clusters (derived from MDA-MB-231-LM2) showed reduced apoptosis compared to solitary cells when injected via tail-vein to colonize the lungs in a pre-clinical model of breast cancer metastasis [5]. Another clear difference between CTC clusters and solitary CTCs is cell-cell adhesion and interaction within the cluster. Many epithelial markers have been detected within CTC clusters that support cell anchoring, desmosome, and hemidesmosome function such as E-cadherin, plakoglobin (gamma-catenin), cytoskeletal keratin -5, -8, and -14 [38–42]. In keeping with this observation, cortical dynamics of actomyosin (myosin IIA) within tumor clusters isolated from pre-clinical models can promote adaptation to fluid shear stress (Fig. 1, Box 3) [38]. In addition to physical changes, CTC clusters isolated from patients show distinct epigenetic remodeling compared to solitary CTCs. For example, CTC clusters isolated from breast cancer patients and *in vivo* models exhibit increased hypomethylation in regions controlling pluripotency genes like OCT4, SOX2, and NANOG compared to solitary CTCs [43]. Other studies in pre-clinical models show stemness signatures such as elevated CD44 expression in CTCs [44, 45]. The distinction between solitary CTCs and CTC clusters is not limited to molecular differences but also to the cellular composition and architecture of CTC clusters. CTC clusters can be either homotypic (comprised of cancer cells only) or heterotypic (mixed with stromal or immune cells) [35]. Heterotypic CTC clusters have been observed to contain non-cancerous cells within the cluster including cancer-associated fibroblasts (CAFs) [46, 47], white blood cells [48] like tumor-associated macrophages (TAMs) [49] and neutrophils [50], platelets [51, 52], and others [53]. Collectively, these molecular and cellular differences indicate that CTC clusters may confer advantages over solitary cells in terms of invasion, survival in circulation, dissemination, and dormancy in metastatic sites.

## CTC cluster invasion

Tumor initiation and progression elicit various changes within the tumor microenvironment (TME), which can lead to an increased intra-tumor heterogeneity and decreased vasculature integrity. Collectively, these changes give rise to the shedding and intravasation of cancer cells to vascular and lymphatic circulation via passive (through vascular patency) or active (via cancer cells with migratory features) mechanisms [54]. The current paradigm explaining the invasion of CTC clusters includes aggregation of solitary CTCs in the vasculature or the collective migration of CTCs using aspects of the epithelial-mesenchymal transition (EMT) program [54, 55].

### Intravascular aggregation of CTCs

Early and current studies on CTC clusters have sometimes identified them as “aggregates” of CTCs that form later in the vasculature rather than at early stages before intravasation [29, 30, 44, 56, 57]. For instance, studies in 1974 demonstrated that cancer cell aggregates of the mammary cancer cell line C3H metastasized more efficiently to the lungs of syngeneic mice after intravenous injection compared to single cells [29]. Similar findings were observed in a pre-clinical model of colon cancer liver metastasis, where aggregated cancer cells displayed significantly higher metastatic efficiency than the equivalent number of single cells [30]. The process of cancer cell aggregation within the vasculature involves sequential physical steps of binding to endothelial surface adhesion molecules and various molecular interactions including cancer surface integrins and extracellular matrix (ECM) degradation [58]. The hypothesis that CTC aggregates form within the circulation has been tested using suspension culture methods to generate cancer cell aggregates. Despite the lack of fluid shear stress and other factors in this approach, cancer aggregates displayed increased metastatic potential when implanted in mice compared to single cells [56]. Attachment of cancer cells to the microvascular endothelium is suggested to be mediated by a sequence of adhesive events governed by T antigen/galectin-3 interactions [57].

It should be noted, however, that the theory of vascular aggregation of solitary CTCs is somewhat controversial. For example, in a mouse model with fluorescently tagged mammary cancer cells (GFP or mCherry), CTC clusters were shown to be a product of tumor aggregates originating in the primary tumor and not intravascularly [5]. In support of this, studies where either GFP or RFP expressing breast cancer cells (MDA-MB-231-LM2) were injected separately into contralateral mammary fat pads of the same animal demonstrated that five weeks after injection, the majority (96%) of CTC clusters isolated from the peripheral blood were of a single color [5]. Similarly, lung metastases derived from separate orthotopically injected mTomato or CFP expressing PyMT-MMTV breast cancer cell lines were composed of single colors, suggesting again that CTC clusters formed prior to entering circulation [39]. Notably, a spontaneous model of pancreatic cancer metastasis using confetti lineage-labeling system showed that polychromatic micrometastases were observed largely in peritoneal and diaphragmatic metastases (~80%), but minimally in liver and lung metastases (11–14%) which mainly consisted of monochromatic metastases [59]. These findings suggest that clusters may contribute differently to metastatic outgrowth depending on the organ site. Furthermore, multicolor-lineage tracing in pancreatic and breast cancer mouse models also confirmed that serial delivery of labeled cancer cells two [39] or three days [59] apart results largely in monochromatic micrometastases suggesting that polyclonal metastases are likely to emerge from multi-cellular seeding rather than aggregation or sequential seeding of solitary cells [5]. In conclusion, CTC clusters are likely the result of collective migration of cancer cells from primary tumor, but aggregation of CTCs may also occur in certain anatomical locations as well.

### Hybrid EMT and collective invasion of CTC clusters

The collective movement of cells can be observed physiologically in keratinocyte cell sheets during wound healing via TGFβ-mediated EMT, which downregulates E-cadherin at the leading edge [60]. Similarly, the collective movement of cancer cells can be promoted by different EMT phenotypes. For instance, the EMT transcription factor Snail has been implicated in maintaining cell-cell contact within CTC clusters of squamous cell carcinoma by inducing the tight junctional protein claudin-11 [61]. Additionally, CTC clusters, derived from epithelial cancers, express cell-surface vimentin (mesenchymal marker) that induces invasiveness and migration in cancer [62, 63]. However, EMT is not a binary process but rather a spectrum of cell phenotypes that allow for adaptation to specific TME contexts [4]. CTC clusters can be induced through a hybrid EMT phenotype, in contrast to complete EMT in solitary cells [37]. The hybrid EMT phenotype combined with epithelial markers enable CTC clusters to maintain cell-cell adhesion and promote collective migration. Previous studies have noted that CTC clusters, from tumors displaying a partial EMT phenotype, maintained expression of E-cadherin at cell-cell junctions, while solitary CTCs from tumors that had completed EMT lacked any E-cadherin expression [40]. Other epithelial markers have also been implicated in maintaining cell-cell interaction in CTC clusters including cytoskeletal keratin 5, 8, and 14 [39, 41, 42]. Plakoglobin, which is important for desmosome and adhesion complex formation, has also been detected in CTC clusters further implicating the epithelial features within these clusters in collective migration [39]. Interestingly, knocking down plakoglobin in lung metastatic breast cancer cells significantly reduced CTC cluster formation and lung metastatic nodules in NOD-SCID gamma (NSG) mice while having no effect on tumor growth [5]. Overall, more studies are required to strengthen our knowledge about CTC cluster invasion such as the use of intravital imaging and assessing cancer cell collective migration next to tumor microenvironment of metastasis (TMEM) doorways [64, 65].

## CTC cluster survival during circulation

Both solitary cells and CTC clusters have been detected in the peripheral blood of cancer patients. However, CTC clusters are detected at a lower frequency in patients, accounting for approximately 2–3% of all CTCs [5, 32]. Despite this, their detection strongly correlates with poor overall survival [5, 32]. In a pre-clinical model of breast cancer, CTC clusters have a shorter circulation half-life (*t*_1/2_ 6–10 minutes) compared to solitary CTCs (*t*_1/2_ 25–30 minutes) [5]. This may be due to the large size and slow velocity of CTC clusters in the circulation, which in turn leads to a higher chance of their lodging in small blood vessels [66]. It is also possible that CTC clusters may be better equipped for preferential seeding in certain niches but the factors that govern how CTC clusters seed different soils remain unknown.

Metastasis is a highly inefficient process and CTCs face rigorous challenges to survive in circulation [67]. In addition to *anoikis* and shear stress, CTCs must also evade immune recognition. More evidence is emerging as to how CTC clusters may have a greater advantage in overcoming these challenges compared to solitary CTCs [37]. For instance, CTC clusters have been shown to be less apoptotic by measuring condensed or fragmented nuclei compared to solitary CTCs using blood filtration approach in lung cancer patients [36]. The higher potential of CTC clusters to overcome *anoikis* has been attributed to several molecules that regulate cell-cell adhesion. For example, interactions between galactoside-binding galectin-3 (in circulation) and the transmembrane mucin protein MUC1 (on CTC aggregates) can protect clusters from *anoikis* through polarization of MUC1 surface localization. The polarization of MUC1 allows other heterotypic cell-cell adhesion molecules to be exposed for interaction like E-cadherin [68]. In addition, CTC clusters were observed to be adaptive to shear stress in circulation in a mouse model of HNSCC through actin/actomyosin cortical dynamic and E-cadherin mediated cell-cell adhesion (Fig. 1, Box 3) [38]. As discussed before, the loss of E-cadherin has been implicated in invasion of cancer cells and metastasis, but the presence of E-cadherin in CTC clusters might suggest a role in preventing *anoikis* in CTC clusters [40, 69]. Additional evidence on epithelial features of CTC clusters preventing *anoikis* is linked to the detection of several epithelial markers in CTC clusters such as plakoglobin, Keratin 14 (K14), desmosome, and hemidesmosome in CTC clusters [41, 42]. Microtentacle formation by CTC clusters has also been implicated in alleviating shear stress and *anoikis*. Microtentacles are membrane protrusions derived from microtubules yet supported by other molecules like vimentin and different forms of tubulin [70, 71]. CTC clusters show higher levels of microtentacles, which enable them to cluster together or with peripheral blood mononuclear cells like macrophages [72, 73]. Using a zebrafish model, CTC clusters were found to reorganize their structure while passing through tiny capillaries reducing the hydrodynamic pressure applied on them to maintain their viability (Fig. 1, Box 4) [74]. Collectively, CTC cluster superiority in survival during circulation compared to solitary CTCs has been noted, and multiple molecular mechanisms have been uncovered. Importantly, the identification of these mechanisms has allowed for the discovery of potential CTC cluster targeting strategies. For example, sodium-potassium pump inhibitors can dissociate CTC clusters derived from breast cancer patients’ CTCs, which suppressed their metastatic potential [43].

## Dissemination of CTC clusters

Once CTC clusters overcome the harsh conditions of circulation, they disseminate to secondary tissues. CTC clusters are larger in volume than solitary CTCs, which make their circulation velocity slower [66]. This increases their chances of margination (being away from the core of the blood vessel lumen (Fig. 1, Box 2)) and attaching to the vascular wall, hence facilitating extravasation from the circulation [7]. Moreover, CTC clusters were shown to adhere faster than solitary CTCs to E-selectin coated plates in an *in vitro* microfluidic system, which could imply an advantage in extravasation [75]. Additionally, Interleukin 6 (IL-6) and tumor necrosis factor-α (TNF α), which are elevated in metastatic breast cancer patients, have been shown to induce the binding of MDA-MB-231 tumor spheroids to E-selectin coated microtube surfaces compared to single cells that failed to achieve such binding [76]. Additional systemic molecules present in blood can promote CTC cluster binding to the endothelium such as galectin-3, which is also elevated in the sera of metastatic cancer patients [68, 77, 78]. The molecular biology behind solitary and CTC cluster dissemination remains elusive due to technical difficulties in modeling the circulation or observing CTC dissemination within the circulation. However, this obstacle can be overcome with early dissemination *in vivo* models of metastasis and advanced intravital imaging.

## Traveling microenvironment within CTC clusters

CTC clusters can contain non-cancerous cells within the cluster, and these may provide additional advantages to their survival and metastatic potential. For example, CAFs in CTC clusters can increase cancer cell viability, while their depletion reduces the number of metastases in a pre-clinical model of lung cancer. Interestingly, the advantage of cluster bound CAFs could not be replicated when cancer cells and CAFs were inoculated together as single cells suggesting that direct association (clustering) prior to entry into the circulation is needed to confer such advantage [47]. As a means of physical protection against shear stress, platelets have been observed to associate with CTC clusters forming thrombi (Fig. 1, Box 3) [3, 51, 55, 79]. Fibrin generated by platelets allows for the adhesion of cancer cells through their surface ICAM-1 to integrin *α*ΙΙb*β*3 on platelets, thus providing protection against *anoikis* in circulation [52]. Platelets have been also implicated in inhibiting the antitumor activity of natural killer cells (NK cells) through a TGFβ-mediated decrease in natural killer group 2, member D (NKG2D) activating immunoreceptor on NK cells [80]. This was also reported for breast cancer as CTC clusters were shown to resist NK cell killing better than solitary CTCs. The resistance to NK cell killing for clusters was due to the upregulation of NK inhibitory ligands such as Qa1^b^ and downregulation of activating ligands such as Ulbp1 [81]. Interestingly, induction of EMT in the breast epithelial MCF10A cell line by TGF-β resulted in upregulation of NK cell activating NKG2D ligands suggesting a protective phenotype of more epithelial cells against NK cell-mediated killing [81]. These findings suggest a role of cluster-bound platelets in inhibiting immune recognition of CTC clusters. Neutrophils have been also observed in association with CTC clusters and can enhance cancer cell cycle progression within the circulation [50], which is in agreement with previous studies showing a mix of proliferating and quiescent cells within the clusters [43, 82]. Notably, neutrophils produce extracellular traps (NETs) during lung-induced inflammation, which promotes breast cancer metastatic seeding and awakening *in vivo* (Fig. 1, Box 5) [83]. In addition to immune cells, other cellular components of the primary tumor microenvironment can provide advantages to CTC clusters during dissemination. For example, CTC clusters containing endothelial cells are hypothesized to have better angiogenic potential upon dissemination [3, 41, 47]. Currently, the role of additional stromal or immune cells within the clusters remains to be studied in the context of extravasation and dissemination.

## Mechanisms controlling dormancy and reawakening of disseminated tumor cells (DTCs)

Upon arrival and extravasation in their new metastatic setting, DTCs can be identified by immune cells and successfully eliminated or survive by avoiding immune recognition. However, in response to their intrinsic qualities or microenvironmental cues within the metastatic site, cancer cells can be induced to become senescent, an irreversible growth arrest state [84], or enter into dormancy (reversible long term growth arrest) [85]. Despite extensive efforts in the past few decades, the precise mechanisms controlling DTC dormancy and more specifically, whether those mechanisms differ in the context of DTC clusters, has not been fully established. Like CTCs, DTCs are found to be solitary or clusters (also known as micrometastasis) [82, 86]. Whether CTC clusters result in micrometastasis or solitary DTCs is yet to be investigated.

One of the most heavily studied pathways in the context of dormancy and reawakening is the TGFβ pathway, in which TGFβ1, TGFβ2, BMP4, and BMP7 can regulate either dormancy or reawakening in a context and niche dependent manner [87, 88]. Moreover, AXL (tyrosine receptor kinase) and its ligand GAS6 have been implicated in promoting dormancy, especially in the bone marrow niche [89–92]. Similar roles have been demonstrated for leukemia inhibitory factor (LIF) and STAT3 [93]. The cell surface and cell-cell interaction mediated by β1 integrin may also promote reawakening of cancer cells [94, 95]. The microenvironment can play additional roles in regulating both dormancy and reawakening [85, 87, 96, 97], including but not limited to mesenchymal stem cells via the expression of TGFβ2 [98], macrophages by promoting dormancy and stemness genes [99, 100], neutrophils through the emission of neutrophil extracellular traps (NETs) [83], CD4 and CD8 T cells through IRF7 [101, 102], osteoblasts through GDF10 [103], and ECM remodeling through matrix metalloproteinases (MMPs) [83, 104]. We will highlight the current efforts to uncover mechanisms of dormancy and reawakening of either solitary or CTC clusters with emphasis on potential areas of future research.

### Cell cycle and circadian mediators

It is important to note that some regulators of senescence overlap mechanistically with dormancy such as p16, p21, and p53 [105]. Dormancy, however, is defined as reversible cell cycle arrest accompanied by survival programs that support long term viability in the metastatic setting [85]. The dormancy program is believed to be responsible for secondary disease or relapse in patients [84, 106]. DTCs can acquire dormancy by different means: cellular dormancy in which cells enter a quiescence program, micrometastasis dormancy in which tumor cells fail to grow due to a proliferation/ death equilibrium described in the immunoediting theory, and angiogenic dormancy where cancer cells fail to grow due to the lack of angiogenic potential [85, 96, 107, 108]. Dormant cancer cells can exit the cellular dormancy program upon receiving intrinsic or microenvironmental signals to resume their cell cycle progress and proliferate, thus establishing macrometastases. If DTCs failed to acquire these molecular programs or are recognized by immune cells, they will likely be eliminated—an occurrence which is hypothesized to happen often, given the inefficiency of metastasis and failure of many DTCs to initiate successful metastases [109]. The acquired dormancy program can last for years with some patients developing metastases more than a decade after remission [110]. Many markers have been linked to the dormancy phenotype either in patients or pre-clinical models. A common noted dormancy marker across the literature is G0/G1 cell cycle arrest, which is controlled through different signals including p38/MAPK pathway that ultimately induces cell cycle inhibitors such as p21 and p27. These cell cycle inhibitors act on cyclin dependent kinases like CDK4 resulting in cell cycle arrest. It is also well accepted that dormant cells are negative or low for proliferation markers like Ki-67, p-Ser10 histone-H (p-H3), and p-retinoblastoma protein (p-Rb S249/T252) [84, 85, 96, 103].

Circadian rhythm has been implicated recently in regulating the intravasation and metastatic potential of CTCs in breast cancer patients and pre-clinical models. Analysis of CTCs from blood samples of patients and several mouse models showed enrichment of CTC abundance during rest phase (4:00 am for patients) compared to active phase (10:00 am) with CTC clusters showing higher fold change compared to solitary cells in four different pre-clinical models of breast cancer [111]. Injecting solitary or clustered CTCs (homotypic or heterotypic), isolated from mice at rest phase, via tail vein into mice also at rest phase shows higher tumor burden in mice injected with CTC clusters compared to solitary cells [111]. This increase in tumor burden can be the result of a faster initiation of proliferation programs, a more robust proliferation program, or a specific trait of CTC clusters controlled by circadian rhythm. However, little is known about the circadian program during early dissemination of CTC clusters including preferential niches, stromal interactions, and mechanisms of dormancy and reawakening.

### Cell adhesion and ECM regulators

Current studies addressing dormancy and reawakening from the perspective of clustered cells are challenged by the experimental limitations of being able to model them. Current models include mammospheres, spheroids, and organoids. Clusters are conventionally formed using modified low attachment culture conditions with growth factor supplements, serum deprivation, Matrigel-coated vessels, fibronectin-coated vessels, or modulating the stiffness of synthetic ECM [104, 112–115]. In the murine D2-HAN (hyperplastic alveolar nodule) model of breast cancer dormancy and metastasis, cell lines were generated from successful lung metastases (D2.A1) and dormant lung micrometastases (D2.0R). Several studies demonstrated that β1 integrin is key for promoting reawakening. For example, forced expression of E-cadherin resulted in downregulation of β1 integrin and dormancy in D2.A1 cells *in vitro* and *in vivo* [94, 116, 117]. These findings agree with other studies on solitary dormant cells highlighting the importance of β1 integrin in the reawakening of metastatic cells [94, 95]. Switching from quiescence to proliferation in D2.A1 cells required fibronectin production and signaling through β1 integrin [94]. Similarly, successful colonization of D2.A1 cells in the lungs of mice was dependent on focal adhesion kinase (FAK) and β1 integrin expression [95]. Furthermore, *ex vivo* analysis of patients’ CTCs that are positive for both β1 integrin and urokinase receptor (uPAR) demonstrates higher sphere formation ability, invasiveness, and proliferation compared to CTCs negative for these molecules [118]. Urokinase receptor (uPAR) is a GPI-anchored cell membrane receptor implicated in promoting urokinase (uPA) proteolytic activity in the ECM [119]. It is also implicated in promoting the switch from dormancy to reawakening by interacting with integrins and activating ERK1/2 signaling [96, 120]. Clinically, uPAR is also found to be expressed by DTCs in the bone marrow of patients with solid tumors [121].

The contribution of the ECM is another emerging area of CTC cluster research. For example, lysyl oxidase like 2 (LOXL2) is an integral enzyme for collagen and elastin biosynthesis and stabilization as it oxidizes lysine residues in collagen or elastin resulting in the crosslinking of these ECM components [122]. In MCF-7 mammosphere 3D cultures with basement membrane extract (BME), LOXL2 induced-expression was shown to promote the reawakening of these spheres by acquiring a cancer stem cell-like and an epithelial to mesenchymal transition (EMT) phenotype. Moreover, expression of LOXL2 in MCF-7 cell line increased the percentage of proliferating metastatic lesions *in vivo* [123]. The EMT activator ZEB1, which is implicated in regulating dormancy and reawakening of cancer cells [124], can upregulate LOXL2 through direct binding to its promoter [125]. However, further validation for ZEB1/LOXL2 signaling is needed in the context of both solitary and clustered dormancy.

Physical properties such as ECM stiffness is recognized as an important regulator of dormancy as well. In mouse models, high ECM stiffness has been shown to promote dormancy of cancer spheroids via a Cdc42-Tet2 epigenetic program that regulates cell cycle inhibitors p27 and p21 causing cell cycle arrest. Knock-out of Cdc42 or blocking its entry to the nucleus resulted in reawakening of dormant melanoma B16 tumor-repopulating clusters in 1,050 Pa fibrin gels, while having no effect on the proliferation of B16 solitary cells in rigid plastic culture [114]. Other studies modulating ECM stiffness showed that cluster size along with stiffness may be determinants of proliferation versus dormancy [126, 127]. For example, a study using biomaterial-based *in vitro* model that mimics brain microenvironment stiffness showed that all breast cancer clusters remained dormant in low stiffness (∼0.4 kPa mimicking normal brain stiffness [128]) irrespective of their size. However, high stiffness (∼4.5 kPa corresponding to metastatic brain tumors [129]) only maintained dormancy when clusters were below 5000 cells [126, 130]. In agreement, mathematical modeling of prostate cancer colonization using a hybrid cellular automata (HCA) model of bone microenvironment showed that colonizing cancer clusters must be within certain size to increase the likelihood of establishing a successful metastasis [131]. The size of CTC clusters in cancer patients is variable ranging from 2 cells and up to 45 cells or more in some cases [3]. However, the effect of cluster size on the successful establishment of metastasis in patients requires further analysis and experimentation.

### Metabolic control

Metabolism is coming under intense focus in understanding cluster dormancy. For example, liver kinase B1 (LKB1) has been shown to maintain the survival of dormant spheroids in ovarian cancer *in vitro* via 5′-AMP-activated protein kinase (AMPK) pathway [132]. LKB1 (gene name: STK11) is critical for the phosphorylation and activation of the AMPK stress response pathway [133]. Transiently knocking down LKB1 with siRNA technology resulted in reduced viability in ovarian cancer cells growing as spheroids but not as adherent cells [132], providing more context to the possible role of LKB1 loss of function in cancer initiation [134]. AMPK acts as a sensor for ATP levels in the cell, promoting the production of ATP through catabolic pathways [135]. AMPK activation has been documented in dormant cells in a breast cancer mouse model of estrogen deprivation [136]. Moreover, Nuclear Factor Erythroid 2 Like 2 (Nrf2) expression has been shown in both residual and recurrent breast cancer *in vivo* [112]. The Nrf2/Keap1 pathway plays a major part in resistance to oxidative stress by maintaining redox homeostasis [137]. Stable knock-down of Nrf2 via shRNA technology resulted in growth impairment of recurrent breast cancer tumor at early time points, while tumors regained Nrf2 expression at later time points highlighting the potential importance of Nrf2 expression in reawakening [138]. These studies highlight metabolic pathways utilized by solitary or clustered cells that potentially could be therapeutically leveraged to target both CTCs and DTCs.

### Stemness gene control of dormancy

The dormancy phenotype requires plasticity by accessing stemness genes to promote long-term survival of quiescent DTCs in distant tissues [84]. Multiple stemness genes have been studied in the context of dormancy including NR2F1, ZEB1, ZEB2, SOX2, SOX9, and ZFP281 [124, 139–142]. A shift toward mesenchymal phenotype in DTCs may correlate with a dormant phenotype. For example, the EMT activator ZEB1 was found to promote expression of stemness transcription factors like SOX2 and KLF4. ZEB1 promotes stemness genes by inhibiting miR203, which suppresses these stemness genes [124]. Moreover, the ZFP281-mediated mesenchymal program was recently shown to induce long term dormancy of early disseminated cancer cells (eDCCs), which are cancer cells escaping the primary tumor early during carcinogenesis [142]. NR2F1, which is an orphan nuclear receptor implicated in lineage differentiation, was found to induce head and neck cancer dormancy by inducing multiple stemness factors like SOX9 and NANOG [139]. Emerging studies are investigating treatment options that target the stemness signature of dormant DTCs like NR2F1 agonists [143]. DNA methylation profiling of solitary CTCs and CTC clusters from patients or mouse models reveals specific CTC cluster signature of hypomethylated sites of stemness that allow binding of transcription factors like OCT4, SOX2, and NANOG [43]. This unique signature was found to be dependent on cell-cell adhesion within CTC clusters. Disrupting cell-cell adhesion by increasing intracellular Ca^+2^ caused DNA methylation remodeling of key stemness genes in CTC clusters, which confers an architecture advantage of CTC clusters over solitary CTCs in acquiring stemness and long-term survival [43]. Furthermore, CD44, a surface adhesion molecule and marker of cancer stem cells, has also been found to mediate tumor cell aggregation in a patient-derived breast cancer model through homophilic interaction and downstream activation of FAK signaling [44]. Similarly, CD44 standard isoform (CD44S), which is upregulated in cancer cells, was found to protect against *anoikis*, while CD44 depletion attenuated mammosphere formation *in vitro* [45]. CUL4B, an E3 ubiquitin ligase required for the proteolysis of different DNA replication regulators, was implicated in maintaining colorectal cancer stemness by downregulating miR34a. Inhibiting CUL4B in patient derived tumor organoids reduced their metastatic capacity and proliferation [144]. Collectively, stemness genes have been implicated in metastasis and been detected in CTCs from cancer patients [145], CTC clusters from patient derived xenografts (PDX) models [146], and colorectal tumor-derived organoids [147]. Consequently, further investigations are required to confirm the biological advantages gained through stemness genes in relation to understanding their contribution to the dormancy of solitary or CTC clusters.

### Immune microenvironment control

Recognition and elimination by surveilling immune cells often protects against the successful establishment of metastases; however, emerging data also suggests that cellular components of the immune system can be co-opted to control dormancy entry and reawakening. For example, in an orthotopic model of breast cancer using GFP and mCherry labeled AT3 cells, the abundance of polyclonal lung metastases (resulting from clustering of GFP and mCherry cells) was not different between immunocompetent, T cell deficient, and T cell and NK cell deficient C57BL/6 mice. Conversely, monoclonal lung metastases (resulting from single color cells) were higher in T and NK cell deficient mice. Interestingly, breast cancer cell lines with an ability to form clusters had higher E-cadherin expression and lower susceptibility to NK cell killing suggesting a role for the retention of epithelial markers in evading immune recognition and a distinct ability of cancer clusters to evade NK cell killing [81].

## Summary and future directions

Recent advances in technology have yielded numerous studies on the identification and interrogation of solitary and clustered CTCs revealing new cancer vulnerabilities [148]. CTCs clearly harbor biological, structural, molecular, and heterotypical differences that can generally be grouped in two categories: solitary CTCs and CTC clusters. CTC clusters are less abundant in the circulation, yet their detection correlates with poor survival in patients [5]. There has been tremendous effort to study differences between solitary and clustered CTCs, with evidence pointing toward the superior efficiency of CTC clusters to disseminate and survive compared to solitary CTCs. This may be due in part to environmental factors (heterotypic nature), physical factors (clustering and cell-cell contact), biological factors (differential effect of circadian rhythm), and molecular factors [32, 35, 64]. However, many areas remain understudied such as investigation of the collective intravasation of CTC clusters possibly through TMEM doorways, the seeding differences among CTCs in different soils, metabolic differences in CTC clusters that enable survival during metastasis, cellular and molecular interactions of CTC clusters in early dissemination phases of metastasis, and investigating the role of different cellular compartments within CTC clusters using *in vitro* coculture methods. Another major opportunity for future research is to uncover the molecular mechanisms controlling dormancy and reawakening of disseminated cells originating from solitary or clustered CTCs. This exploration will fuel therapeutic strategies to awaken DTCs then eliminate them during treatment of the primary disease, manipulate DTCs to remain dormant in distant tissues, or ultimately eliminate DTCs during initial treatment rounds, hence extending patient disease free survival and preventing relapse.
